# A Nationwide multicenter registry and biobank program for deep phenotyping of idiopathic and hereditary pulmonary arterial hypertension in Korea: the PAH platform for deep phenotyping in Korean subjects (PHOENIKS) cohort

**DOI:** 10.1186/s40885-019-0126-8

**Published:** 2019-09-15

**Authors:** Albert Youngwoo Jang, Sungseek Kim, Su Jung Park, Hanul Choi, Pyung Chun Oh, Seyeon Oh, Kyung-Hee Kim, Kye Hun Kim, Kyunghee Byun, Wook-Jin Chung, for the PHOENIKS Investigators, Wook-Jin Chung, Kyung-Hee Kim, Kye Hun Kim, In-Cheol Kim, Gee Hee Kim, Gi-Beom Kim, Jae-Hyeong Park, Jung-Woo Son, Jong-Min Song, Sang Jae Rhee, Ju-Hee Lee, Jo Won Jung, Hae Ok Jung, Goo-Yeong Cho, Jeong Hyun Choi, Sun-Hwa Lee, Soo Yong Lee

**Affiliations:** 1 Gachon Cardiovascular Research Institute, College of Medicine, Gachon University, Incheon, South Korea; 2 Division of Cardiovascular Disease, Department of Internal Medicine, Gachon University Gil Hospital, Medical Center, Incheon, South Korea; 3 Center for Genomics and Proteomics, Institute for Regenerative Medicine, Lee Gil Ya Cancer and Diabetes Institute, Gachon University, Incheon, South Korea; 4 Department of Cardiology, Sejong General Hospital, Bucheon, South Korea; 5 Department of Cardiology, Chonnam University Hospital, Gwangju, South Korea; 6 Department of Cardiovascular Medicine, School of Medicine, Gachon University, 21 Namdong-daero 774beon-gil, Namdong-gu, Incheon, 21565 Republic of Korea

**Keywords:** Pulmonary arterial hypertension, Precision medicine, Blood bank, registries

## Abstract

**Background:**

Pulmonary arterial hypertension (PAH) is a progressive, chronic disease without curative treatment. Large registry data of these patient populations have been published, although, phenotypic variants within each subtype of PAH have not been elucidated. As interest towards personalized medicine grows, the need for a PAH cohort with a comprehensive understanding of patient phenotypes through multiomics approaches, called deep phenotyping, is on the rise. The PAH Platform for Deep Phenotyping in Korean Subjects (PHOENIKS) cohort is designed to collect clinical data as well as biological specimens for deep phenotyping in patients with idiopathic PAH (IPAH) and heritable PAH (HPAH) in Korea.

**Methods:**

A total of 17 regional hospitals are currently working on enrolling up to 100 consecutive IPAH/HPAH patients for obtaining clinical data and biological specimens across Korea. The diagnosis of PAH is based on right heart catheterization. All clinical data is stored in a government-based online database. Each participating hospitals collect a whole blood sample from each patient, through which DNA, RNA, serum, plasma, and peripheral blood mononuclear cells will be extracted from the buffy coat layer for further multiomics analysis.

**Results:**

Not applicable.

**Conclusions:**

The PHOENIKS cohort is enrolling IPAH and HPAH patients across Korea to determine the prognosis and drug response in different phenotypic variant. The data generated by this cohort are expected to open new doors for personalized medicine in PAH patients of South Korea.

**Trial registration:**

ClinicalTrials.gov NCT03933579. Registered on May 1st, 2019.

## Introduction

Pulmonary hypertension (PH) is defined as a mean pulmonary artery pressure ≥ 25 mmHg, while pulmonary arterial hypertension (PAH) is one of the five categories of PH classified by World Health Organization (WHO) [1]. PAH is precapillary pulmonary hypertension caused by vasoconstriction and proliferative remodeling of precapillary arteries resulting in increased pulmonary vascular resistance, right ventricular heart failure, and eventually, death [2]. Although previously published large scaled registries have substantially improved our understanding of PAH [3–6], these data lack deep phenotyping, which is, gathering the detailed genetic and molecular information that underlies the prognosis or response to specific drugs of each patient [7–12]. Current registries, such as the National Biological Sample and Data Repository for PAH and the Biomedical Research Identification of Genetic Etiology of PAH (BRIDGE-PAH), are actively recruiting patients in the US and UK, respectively, and are aiming for deep phenotyping of PAH patients [12, 13]. These trends give rise to the need for an East Asian population-based PAH registry that incorporates deep phenotyping as well as robust genetic and multiomics level data from PAH patients. The PAH Platform for Deep Phenotyping in Korean Subjects (PHOENIKS) cohort aims to build a database based on clinical data and biospecimens for PAH patients across South Korea.

## Methods

### Study objectives

The current study is a multicenter registry and biobank enrolling South Korean populations to construct a database for the elucidation of molecular and genetic modifiers of PAH. The main goal of the current study is to evaluate clinical outcomes in South Korean PAH subjects and collect biospecimens for subsequent deep phenotypic studies. Detailed study objectives are shown in Table 1. Specific goals regarding deep phenotyping will be described in subsequent studies.

**Table 1:** Study objectives of the cohort

Study Objectives	
1. To build a robust IPAH and HPAH patient registry through collaborative network across South Korea with 16 regional hospitals across the country.	
2. To perform a long-term follow up of registered patients to characterize the clinical course of IPAH and HPAH patients among South Koreans.	
3. To collect biospecimens of enrolled patients and to build a biobank for Korean PAH and HPAH.	
4. To identify novel genetic mutations via next generation sequencing of the collected biospecimens.	
5. To deep-phenotype PAH and HPAH through a detailed omics-level study from a large collection of patient samples.	

*IPAH* idiopathic pulmonary arterial hypertension, *HPAH* hereditary pulmonary arterial hypertension

### Study sample

The current study will initially exclude the most frequent form of PAH in South Korea, i.e., connective tissue disease (CTD)-related PAH [5], and focus on the diagnosis and deep phenotyping of idiopathic PAH (IPAH) and heritable PAH (HPAH) patients. The enrolling 17 tertiary centers within South Korea are as follows - Gachon University Gil Medical Center, Incheon; Sejong General Hospital, Bucheon; Chonnam University Hospital, Gwangju; Keimyung University Dongsan Medical Center, Daegu; The Catholic University of Korea St. Vincent’s Hospital, Suwon; Seoul National University, Seoul; Chungnam National University Hospital, Daejeon; Wonju Severance Christian Hospital, Wonju; Asan Medical Center, Seoul; Wonkwang University Hospital, Iksan; Chungbuk National University Hospital, Cheongju; Yonsei University Severance Hospital, Seoul; The Catholic University of Korea Seoul St. Mary’s Hospital, Seoul; Seoul National University Bundang Hospital, Bundang; Pusan National University Hospital, Pusan; Chonbuk National University Hospital, Jeongju; Pusan National University Yangsan Hospital, Yangsan. Patients are being enrolled through January 1st, 2018 and December 31st 2021. The inclusion criteria are as follows: (1) over 18 years of age, (2) mean pulmonary arterial pressure of 25 mmHg or higher confirmed by right heart catheterization (RHC), (3) pulmonary vascular resistance ≥240 dynes∙s∙cm^− 5^, and (4) left ventricle diastolic pressure (LVDEP) or pulmonary capillary wedge pressure (PCWP) ≤ 15 mmHg. Exclusion criteria are: (1) patients with drug-induced-PAH; (2) CTD, human immunodeficiency virus (HIV) infection, portal hypertension, congenital heart disease, or schistosomiasis-associated-PAH; (3) long-term responders to calcium channel blockers; and (4) PAH patients with overt features of venous capillary involvement, leaving IPAH and HPAH patients among group 1 of PH to be evaluated [14]. HPAH will be diagnosed by identifying patients with heterozygous pathogenic variants of predetermined genes such as *BMPR2, ACVRL1, ENG, CAV1, SMAD1, SMAD4, SMAD9, KCNK3,* and *EIF2AK4.* Patients without a specific genetic mutations will then be categorized as IPAH patients. The genomic data of family members across 3 pedigrees of an HPAH patient will also be analyzed. All clinical and biological data will be reported to a customized web-based case report form called the iCReaT system managed by the Korean Center for Disease Control. Although the current analysis is planned to be limited to IPAH and HPAH patients, our steering committee plans to expand to all types of PAH in subsequent studies. Among the estimated 1500 total patients of PAH in South Korea, our goal is to enroll 100 consecutive patients.

### Baseline data, clinical outcomes, and biospecimen collection

The baseline data from the registered patients across the 17 regional hospitals will include the following: WHO functional classification, 6-min walking tests, blood samples, electrocardiograms, chest X-rays, echocardiography, optional pulmonary-cardio exercise tests, optional cardiac MRI, RHC, and the evaluation of comorbidities. Detailed information for each exam is displayed in Table 2. The registered patient will be followed up on a regular basis for further data and biospecimen collection. Mortality and hospitalization is planned to be tracked for clinical outcomes.

**Table 2:** Clinical data entries of the cohort

Parameter	Specific categories
Identification Data	Patient code, Date of birth, Registered date
	Sex, Postal code, Height & Weight
Onset	Year of first symptom/first hospital visit/PAH diagnosis
WHO functional class at diagnosis	Class I-IV
Symptoms at diagnosis	Dyspnea/Chest pain/
	Syncope/Edema/Palpitation/Weakness/Other(specify)
Signs at diagnosis	Cardiac murmur/Hepatomegaly/Elevated JVP/Pitting edema/Ascites
Vital sign at diagnosis	Blood pressure-Heart rate-Respiratory rate-Body temperature
PAH family history	Y/N, Specify
Medical history	Connective tissue disease, Congenital heart disease
	PAH inducing drug history, HIV diagnosis/treatment history, Chronic hepatic disease
6MWT	Distance (meters), Blood pressure (before & after),
	Pulse rate, O_2_ Saturation
Blood Chemistry and Hemodynamics	NT-proBNP, troponin I, PT, Cr,
Electrocardiogram	Normal, RVH, nonspecific
Imaging studies	Chest radiography, Echocardiography, Computed chest tomography, Cardiac MRI
Cardiopulmonary exercise test	pCO2, VE/VCO_2_, VO_2_/HR, peak VO_2_
RHC	Systolic, diastolic, mean pulmonary arterial pressure, pulmonary wedge pressure, right heart pressure, central vein, S_v_O_2%,_ Pulmonary vascular resistance

*PAH* pulmonary arterial hypertension, *HIV* human immunodeficiency virus, *6MWT* 6-min walk test, *NT-proBNP* N-terminal prohormone of brain natriuretic peptide, *PT* prothrombin time, *Cr* creatinine, *RVH* right ventricular hypertrophy, *MRI* magnetic resonance imaging, *RHC* right heart catheterization

With the patient’s blood sample, DNA, RNA, serum, plasma, and peripheral blood mononuclear cells (PBMC) from the buffy coat will be separated and be extracted for further storage and studies. For the DNA sample, 2.5 ml whole blood will be stored in a DNA tube (PAXgene® Blood DNA Tube, BD Science, San Jose, CA) at each enrolling center, and it will be transported to the main center at 4–10 °C. At the main center, it will then be transferred to a 2-ml cryotube to be stored at − 70–80 °C. For RNA collection, 2.5 mL whole blood will be collected in PAX gene RNA tubes, and will be sent to the main center at 4–10 °C, where it will also be further transferred to a cryotube to be stored at − 70–80 °C.

Serum, plasma and the buffy coat will also be collected. At the respective collection sites, serum will be collected in serum separation tubes, and 30 min after venipuncture, it will be centrifuged and stored at − 20 °C. For plasma, cell preparation tubes will be used within 2 h of blood collection. Then, each sample will be ready to be stored at − 20 °C by treating the samples with human serum type antibody, DMSO, and freezing medium. To separate and extract PBMC, the white buffy coat layer will be separated and be stored in a 1.5-ml tube with freezing medium at − 20 °C. The collected baseline data will then go through a clean-up process and further evaluation of its quality will be done. In addition, five patients will be selected to perform whole-genome sequencing. Half the sample of all collected biospecimens will be stored at the Korean National Institute of Health main storage and the other half at Gachon Cardiovascular Research Institute at Gachon University. Biospecimens will be further used for multiomics studies and subsequently deep phenotyping. Specific methods regarding multiomics deep phenotyping is beyond the scope of this paper and will be dealt with in the subsequent study.

### Patient follow-up

All patients will be followed up twice or more per year, with an expected 80% follow-up rate. During the second and third year, patient registry and data collection will be continued using the same protocol. To maintain the credibility of collected data, the steering committee will continuously monitor and audit the collected data. The protocol may be further crafted after evaluating the previous year’s data. Effective data management strategies, including a guideline to standardize body measurements and blood samples, will be developed. Ten patient samples will be selected for next generation sequencing and will be utilized to discover novel genetic mutations.

### Genetic mutation analysis across three-pedigrees for HPAH patients

To determine patients with HPAH, a familial genetic study will be performed. A three-generation pedigree for each PAH patient with or without the existence of *BMPR2, ACVRL1, ENG, CAV1, SMAD1, SMAD4, SMAD9, KCNK3,* and/or *EIF2AK4* mutations will be evaluated. Blood samples from family members of patients with a HPAH will be collected for genetic screening.

### Statistical analysis

Continuous normally-distributed data will be expressed as the means ± standard deviations. Student’s t-tests or one-way ANOVA tests will be used to compare inter-group differences for normally distributed variables. Categorical variables will be analyzed using Pearson’s χ^2^ test or Fisher’s exact test. For all analyses, a two-sided *p* < 0.05 will be considered statistically significant. For longitudinal data comparisons, we will use the Mantel-Cox method to calculate hazard ratios and 95% CIs and the log-rank test to calculate corresponding *p* values. Data analysis will be performed using IBM SPSS Statistics (IBM Corp. Released 2014. IBM SPSS Statistics for Windows, Version 20.0. Armonk, NY: IBM Corp.). Due to the small patient population and seldom references for sample size calculation, the current project will be enrolling 100 consecutive PAH patients initially. Sample size expansion will be done in subsequent studies.

## Results

Not applicable.

## Discussion

A high degree of phenotypic variability within IPAH and HPAH has been increasingly recognized, bringing into question the efficacy of the current PAH treatment that uniformly targets the classical PAH pathways [15, 16]. Such heterogeneity has resulted in variable treatment responses among patients. Accordingly, it is necessary to develop better methods for personalized medicine - predicting responses of different classes of PAH medications in each individual. Although, personalized medicine is a familiar concept that is already used in PAH, as patients with vasoreactivity with nitric oxide are treated with calcium channel blockers, better understandings of the response to treatment in different cellular and molecular backgrounds is necessary for a more precise prediction [8]. Deep phenotyping is a multiomics approach to understand the cellular and molecular processes that underlie the variability to each treatment. Using a multiomics approach allows the acquisition of large amounts of data from gene sequencing and proeomic analysis to metabolic profiling [7]. Accordingly, personalized medicine can be achieved only through the foundation of deep phenotyping. Through such data, the mechanisms underlying the phenotypic variability in treatments or type of PAH may be understood [9, 10, 17–20]. We expect that the large datasets collected by the PHOENIKS project will cover many novel molecular-level mechanisms that may explain the heterogeneous phenotypes among South Korean individuals.

Previous registries in Europe, North America and Asia have focused on the clinical aspects of the disease [3–6, 21], providing better understandings of the prevalence of each PAH subtype and prognosis. Such data, however, were not designed to elucidate the gaps in our understandings of deep phenotyping [22]. The aims of the PHOENIKS cohort share similarities with those of the new BRIDGE-PAH registry in the UK and the National Biological Sample and Data Repository for PAH in the US [12, 13]. Both studies are currently actively recruiting patients and are expected to elucidate the molecular pathobiology of PAH by a multiomics approach. To our knowledge, the PHOENIKS cohort will be the first PAH registry acquiring deep phenotypic data in all of Asia.

Because PAH is a rare disease, a collaborative effort for building the patient database is essential. The current registry is the first collaborative network for deep phenotyping of PAH patients across South Korea. Through this network, the pipeline for collecting clinical data and biospecimens will be standardized, thereby founding a solid basis for further research. Furthermore, the groundwork done by PHOENIKS is expected to open doors for finding novel candidate molecules for potential therapeutic targets. PAH is a complex disease caused by the intertwined effects of several factors and conditions, including genetic, environmental, and inflammatory factors [23]. Selected candidate molecular functions and mechanisms related to the disease can be then analyzed via in vitro and in vivo assays.

## Conclusion

The recent growing interest towards personalized medicine urges the need for deep phenotyping in the field of PAH. The PHOENIKS cohort is expected to register up to 100 IPAH and HPAH patients across Korea to identify the relationship between phenotypic variants from genetic expression to protein translation and metabolites, responses to classical drugs and prognosis. The data generated by this cohort are expected to open new doors for precision/individualized medicine in Korean PAH patients.

## References

[1] Hoeper MM (2013). Definitions and diagnosis of pulmonary hypertension. J Am Coll Cardiol.

[2] Hoeper MM (2016). A global view of pulmonary hypertension. Lancet Respir Med.

[3] Hoeper MM (2013). Elderly patients diagnosed with idiopathic pulmonary arterial hypertension: results from the COMPERA registry. Int J Cardiol.

[4] McGoon MD, Miller DP (2012). REVEAL: a contemporary US pulmonary arterial hypertension registry. Eur Respir Rev.

[5] Chung WJ (2015). Baseline characteristics of the Korean registry of pulmonary arterial hypertension. J Korean Med Sci.

[6] Jing ZC (2007). Registry and survival study in chinese patients with idiopathic and familial pulmonary arterial hypertension. Chest.

[7] Delude CM (2015). Deep phenotyping: the details of disease. Nature.

[8] Rich S, Brundage BH (1987). High-dose calcium channel-blocking therapy for primary pulmonary hypertension: evidence for long-term reduction in pulmonary arterial pressure and regression of right ventricular hypertrophy. Circulation.

[9] Rhee RL (2015). Comparison of treatment response in idiopathic and connective tissue disease-associated pulmonary arterial hypertension. Am J Respir Crit Care Med.

[10] Sweatt AJ (2019). Discovery of distinct immune phenotypes using machine learning in pulmonary arterial hypertension. Circ Res.

[11] Hemnes AR (2016). Critical genomic networks and Vasoreactive variants in idiopathic pulmonary arterial hypertension. Am J Respir Crit Care Med.

[12] Graf S, Bleda M, Haddinapola C, Haimel M, Bogaard HJ, Coglan G, Corris PA, Gibbs JS, Humbert M, Kiely DG, Laurie A, Machado RD, Peacock AJ, Pepke Zaba J, Toshner M, Trembath RC, Noordegraaf AV, Wharton J, Wilkins M, Wort SJ, Morrell NW, and on behalf of the NIHR BioResource – Rare Diseases(BRIDGE) PAH Consortium , and the UK National PAH Cohort Study Consortium, Whole Genome Sequencing in Idiopathic and Familial Pulmonary Arterial Hypertension Reveals Causal Rare Coding and Non-coding Sequence Variation. in: vascular disease session title: dickinson w. richards memorial lecture. Circulation, 2018. https://www.ahajournals.org/doi/abs/10.1161/circ.134.suppl_1.14413. Accessed 10 July 2019.

[13] Nichols WC, Pauciulo MW, Lutz K, Winslow C, Walsworth A, Gygi A, Marsolo K, Harley JB, Martin LJ *National Biologic Sample and Data Repository for PAH Update.* in: c58. Clinical pulmonary vascular disease. Am J Respir Crit Care Med, 2015. https://www.atsjournals.org/doi/abs/10.1164/ajrccm-conference.2015.191.1_MeetingAbstracts.A4811. Accessed 10 July 2019.

[14] Simonneau Gérald, Montani David, Celermajer David S., Denton Christopher P., Gatzoulis Michael A., Krowka Michael, Williams Paul G., Souza Rogerio (2019). Haemodynamic definitions and updated clinical classification of pulmonary hypertension. European Respiratory Journal.

[15] Humbert M (2010). Survival in patients with idiopathic, familial, and anorexigen-associated pulmonary arterial hypertension in the modern management era. Circulation.

[16] Ling Y (2012). Changing demographics, epidemiology, and survival of incident pulmonary arterial hypertension: results from the pulmonary hypertension registry of the United Kingdom and Ireland. Am J Respir Crit Care Med.

[17] Benza RL (2015). Endothelin-1 pathway polymorphisms and outcomes in pulmonary arterial hypertension. Am J Respir Crit Care Med.

[18] Rhodes CJ (2017). Plasma metabolomics implicates modified transfer RNAs and altered bioenergetics in the outcomes of pulmonary arterial hypertension. Circulation.

[19] Li M (2016). Metabolic Reprogramming Regulates the Proliferative and Inflammatory Phenotype of Adventitial Fibroblasts in Pulmonary Hypertension Through the Transcriptional Corepressor C-Terminal Binding Protein-1. Circulation.

[20] Caruso P (2017). Identification of MicroRNA-124 as a major regulator of enhanced endothelial cell glycolysis in pulmonary arterial hypertension via PTBP1 (Polypyrimidine tract binding protein) and pyruvate kinase M2. Circulation.

[21] Lau EMT (2017). Epidemiology and treatment of pulmonary arterial hypertension. Nat Rev Cardiol.

[22] Savale Laurent, Guignabert Christophe, Weatherald Jason, Humbert Marc (2018). Precision medicine and personalising therapy in pulmonary hypertension: seeing the light from the dawn of a new era. European Respiratory Review.

[23] Satoh Kimio, Kikuchi Nobuhiro, Satoh Taijyu, Kurosawa Ryo, Sunamura Shinichiro, Siddique Mohammad, Omura Junichi, Yaoita Nobuhiro, Shimokawa Hiroaki (2018). Identification of Novel Therapeutic Targets for Pulmonary Arterial Hypertension. International Journal of Molecular Sciences.

